# Prospecting for yaws in the Mbaïki Health District in the Central African Republic

**DOI:** 10.11604/pamj.2023.45.121.34060

**Published:** 2023-07-12

**Authors:** Germain Piamale, Romaric Ghislain Zarambaud Bohy-Ngombet, Rodrigue Herman Doyama-Woza, Emmanuel Fandema, Christian Maucler Pamatika, Constantin Juvénal Dombeti, Henri Saint Calvaire Diemer, Jean De Dieu Longo, Gérard Gresenguet

**Affiliations:** 1 Doctoral School of Human and Veterinary Health Sciences, University of Bangui, Bangui, Central African Republic,; 2 Department of Public Health, Faculty of Health Sciences, University of Bangui, Bangui, Central African Republic,; 3 Integrated Disease Surveillance Service, Bangui University Hospital Center, Bangui, Central African Republic,; 4 Laboratory Department, Maman Elisabeth Domitien University Hospital Center, Central African Republic

**Keywords:** Yaws-prevalence, Mbaïki Health District, Central African Republic

## Abstract

**Introduction:**

yaws is endemic in the Central African Republic. The last cases of yaws notified by CAR to WHO date back to 2012. The objective of this study was to measure the prevalence of yaws in the health district of Mbaïki and to describe its clinical and epidemiological characteristics.

**Methods:**

this is a descriptive cross-sectional study, conducted from April 10 to 18, 2020 in the Mbaïki health district. Yaws cases were sought in 570 households in the 38 selected villages of the district. Any consenting individual over the age of one year with yaws-like skin lesions was a suspected case of yaws and included in the study. Blood was taken from suspected cases for serological testing (TDR, RPR and TPHA). Any suspected case of yaws with positive RPR and TPHA was considered a confirmed case.

**Results:**

a total of 1967 people were examined, of whom 113 were considered suspected cases of yaws. All suspected cases were RPR-positive, 41 TPHA-positive and 13 RDT-positive. Forty-one cases of yaws were confirmed in 18 (47.37%) villages. The prevalence of yaws in the Mbaïki health district was 2.08%. Among the cases, 38.94% were children aged 1 to 14. The sex ratio was 1.69. Lesions clinically suggestive of yaws were papilloma-like in 77.00% of cases, followed by micropapules (8.00%) and ulcerations (5.00%).

**Conclusion:**

eight of the nine communes in the Mbaïki health district are yaws-endemic. This result suggests the need to implement the Morges strategy in the Mbaïki health district.

## Introduction

Yaws, an infectious disease caused by *Treponema pallidum pertenue*, is the most common of the three endemic treponematoses (yaws, bejel and pinta or carate). It is on the list of neglected tropical diseases published by the World Health Organization [1, 2]. Yaws is rife in underprivileged communities in the hot and humid tropical forest regions of Africa, Asia, Latin America and the Pacific, and predominantly affects children under the age of 15 [3]. Yaws is spread by direct skin contact between an infected person and a healthy person through exudate or serum from lesions (papules, papillomas, ulcers) from recent cases [1-3]. The spread of yaws is facilitated by overcrowding, poor collective sanitation conditions, and poor personal hygiene [1-3]. Untreated, yaws evolve in several phases: a primary phase marked by clinical manifestations or active yaws, a secondary phase or latency phase where the infected person does not present any clinical sign, and a tertiary phase during which, Treponema causes cutaneous or osteo-articular damage (bone deformation, mutilation, destruction of the nasal pyramid and chronic disability) [1-3].

From 1952 to 1964, thanks to mass treatment campaigns with benzathine benzylpenicillin administered to patients and their contacts, the number of cases of yaws declined sharply with a reduction in the prevalence of about 95%, from 50 million to 2.5 million cases [4]. But, insufficient resources and sustained political commitment have slowed the progress of the campaign to eradicate the disease [4]. In recent years, we have witnessed a re-emergence of the yaws [5-7]. According to recent estimates, 62,784 suspected cases of yaws were reported worldwide during the period 2014-2016, which corresponds to an average of 20,928 cases per year [8]. The Central African Republic (CAR), a country known to be endemic for yaws [9-14], reported in 2008 and 2012, respectively, 243 and 230 cases of yaws [15]. All of these cases come from two health districts in the country: Mbaïki district and Sangha-Mbaéré district. The objective of this study is to measure the prevalence of yaws in the Mbaïki health district and to describe its clinical and epidemiological aspects.

## Methods

**Figure 1:**
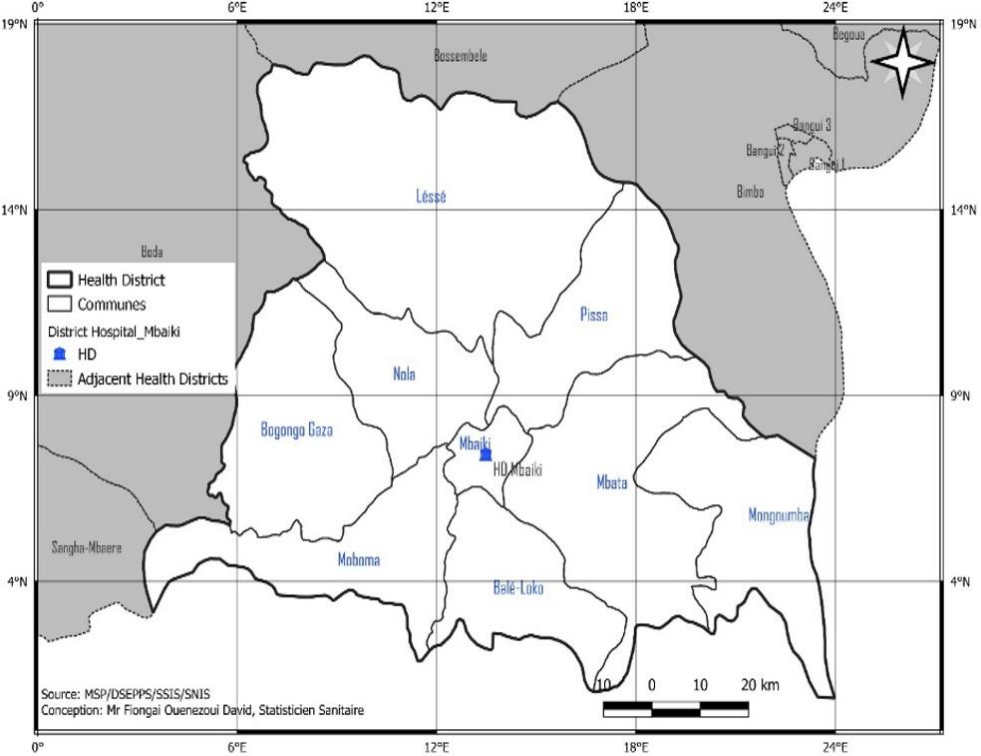
map of the Mbaïki health district

**Study framework:** this descriptive cross-sectional study took place from April 10 to 18, 2020 in the Mbaïki health district. The health district of Mbaïki is located in the south-west of the Central African Republic, 108 km from Bangui, the capital (Figure 1). It includes two sub-prefectures (Mbaïki and Mongoumba), nine communes and 298 Villages. The population served by the health district is estimated at 212,347 inhabitants in 2020, according to projection data from the 2003 General Population and Housing Census.

### Study design

**Sample size:** the sample size was determined by the formula below:


$$n=DEFF\times \left[\left(Z^{2}\times p\left(1-p\right)/C^{2}\right)\times e\right]$$

Where: n: sample size; DEFF: expresses the effect of the sampling plan = 2.653; Z: the standard deviation corresponding to 95% confidence intervals = 1.96; P: expected prevalence = 15%; C: the margin of error = 3%; e: inflation coefficient allowing non-responses to be taken into account =1.2. All calculations considered, the sample size to be surveyed in the health district is estimated at 1,732 individuals. Taking into account the survey methodology adopted, the sample size to be examined and the average number of people per household estimated at three according to the results of the SMART 2019 survey, we have estimated that around 570 households will be needed, spread across 38 villages, and therefore an average of 15 households per village.

### Recruitment of participants

The methodology adopted for this study is that of two-stage cluster sample surveys. In this study, a cluster corresponds to a village or neighborhood. At the first stage of drawing, a sample of 38 villages was selected for the study. The selection of villages was done using the list of 298 villages and neighborhoods of the health district, established based on census cartography data from the 2003 general population census (RGPH). The villages are randomly selected, proportional to their size (PPT) in the health district using the random number generator. At the second stage, after selecting a village or neighborhood, a sample of 15 households is selected by simple systematic sampling. For the draw of households, we established in each selected village, the list of all households, we assigned to each household, a number, which corresponds to the name of the head of household. In the presence of all the household heads of the village and the village chief, we proceeded to the selection of 15 households using the random number generator. In each of the selected households, and after informed consent of the head of household or his representative, a questionnaire was administered to the head of household. Information on: the type (Bantu or indigenous Aka), the lifestyle of the household (sedentary or nomadic), the composition of the household, the presence or absence of each member of the household, occupation for persons over age 14, school attendance for children aged 5 to 14, availability of sources of drinking water and latrines, availability and use of soap, access to health services and use of health care In the event of illness, socio-demographic information on each member of the household was collected. In each household, all members present were examined for skin lesions. Any household member, with a skin lesion compatible with yaws, had blood drawn for an SD Bioline Syphilis 3.0 rapid diagnostic test, the non-treponemal rapid plasma reaction (RPR) flocculation test and the Treponema Pallidum Hemaglutination Assay (TPHA) test for biological confirmation of yaws.

**Inclusion, exclusion and non-inclusion criteria:** were included in the study, any individual over the age of one year, with or without notion of stay in an endemic area, presenting dermatological lesions suggestive of yaws and who gave their consent to participate in the study. Were excluded, individuals more than one-year-old, who did not present any skin lesion. Not included were members of households whose head of household did not consent, children under the age of one, as well as members of households absent when the interviewers visited.

**Data collection:** data collection was carried out by trained interviewers, using smartphones in which an electronic version of the questionnaire was configured, using ODK (Open Data Kit) technology. The questionnaire comprised three modules: the “household” module, the “children aged 1 to 14” module and the “people over 14” module. In each household, the questionnaire was administered to the head of household or his/her representative. All household members were then examined for skin lesions compatible with yaws. Any individual presenting a skin lesion suggestive of yaws was given 5 ml of blood. Three serological diagnostic tests were performed on each sample: the SD Bioline Syphilis 3.0 rapid diagnostic test, the non-treponemal Rapid Plasma Reaction (RPR) flocculation test and the Treponema Pallidum Hemagglutinations Assay (TPHA).

**Data analysis:** data collected on ODK was exported to CSPro, then to Microsoft Excel 2010 for cleaning. After cleaning, the data was exported to SPSS version 22 for analysis. For qualitative variables (presence of skin lesion, location of lesions, number of lesions, type of lesion, etc.), the proportions were calculated. For quantitative variables, means, mode and standard deviation were calculated. Chi^2^ tests were performed to measure the degrees of association between the presence of skin lesion and age or sex.

### Operational definitions

**Suspected case of yaws:** was considered a suspected case of yaws, any individual of any age who is or lived in an already endemic region, presenting clinical signs compatible with yaws (papilloma, ulcer, papule, and macule).

**Confirmed case of yaws:** was considered a confirmed case of yaws, any clinically suspected infectious case (suspected case) which is confirmed by a serology positive for both TPHA and RPR.

**Endemic village:** was considered an endemic village of yaws, a village containing at least one confirmed indigenous case of yaws.

**Clinical prevalence:** number of suspected cases of yaws compared to the number of people examined multiplied by 100.

**Serological prevalence:** number of cases of serologically confirmed yaws compared to the number of suspected cases of yaws multiplied by 100.

**Prevalence of yaws:** number of confirmed cases of yaws compared to the number of people examined multiplied by 100.

**Commune:** grouping of at least 10 villages or neighborhoods.

**Community:** grouping of several members of a household, sharing the same living conditions.

**Ethical and deontological considerations:** the study was authorized by the Ministry of Population and Health of the CAR, and approved by the Scientific Committee for the Validation of Study Protocols and Results of Health Research of the University of Bangui **N°16/UB/FACSS/CSCVPER/19**. All successful participants gave their consent after a detailed explanation of the study objectives. A code was assigned to each of the participants in order to guarantee the confidentiality of the data. The study was carried out in accordance with the Declaration of Helsinki.

**Source of funding:** this study was carried out thanks to a grant offered by the Coordination Organization for the Fight against Endemics in Central Africa (OCEAC), on the basis of financial cooperation between CEMAC and the Ministry of Economic Cooperation and Development (BMZ) of the Federal Republic of Germany, through the KfW (German Development Bank).

## Results

### Characteristics of selected households

Out of 570 planned households, 550 (96.49%) were visited by the interviewers. Of the 550 households visited, only 460 (80.17) heads of household gave their consent to participate in the survey. A total of 79 (14.36%) heads of household and members of their households were absent, and 11 (2.00%) heads of household refused to take part in the survey. Table 1 shows the distribution of villages and households surveyed and the response rate by commune. The majority of heads of household (n=349, 75.87%) were male, with a sex ratio of 3.14. The ages of heads of household ranged from 17 to 80 years, with an average age of 42.45±13.604. Almost half (49.9%) of heads of household were aged between 25 and 44. Household populations surveyed were predominantly Bantu (n=405, 88.04%) with a sedentary lifestyle (n=440, 95.65%). Boreholes (n=231, 50.22%) were the main source of drinking water for households, followed by unprotected water sources (n=145, 31.52%). Only three communes in the district had an access rate to a safe drinking water source ≥ 60%. These were the communes of Lessé (100.00%), Pissa (68.70%) and Mongoumba (64.80%).

**Table 1:** distribution of villages and households surveyed and response rate by commune

Communes	Number of villages surveyed	Number of villages planned	Households visited		Households surveyed		Households absent		Refusals	
			n	%	n	%	n	%	n	%
Mbaïki	3	45	45	100.00	41	91.11	3	6.67	1	2.22
Mbata	6	90	87	96.67	77	88.51	11	12.64	2	2.30
Pissa	6	90	90	100.00	67	74.44	21	23.33	2	2.22
Baléloko	6	90	84	93.33	65	77.38	23	27.38	2	2.38
Moboma	4	60	58	96.67	53	91.38	7	12.07	0	0.00
Nola	4	60	57	95.00	53	92.98	5	8.77	2	3.51
Bogongo-gaza	2	30	29	96.67	27	93.10	3	10.34	0	0.00
Lessé	1	15	15	100.00	6	40.00	9	60.00	0	0.00
Mongoumba	6	90	85	94.44	71	83.53	17	20.00	2	2.35
**HDM**	**38**	**570**	**550**	**96.49**	**460**	**83.64**	**99**	**18.00**	**11**	**2.00**

The majority of households surveyed (n=296, 64.35%) used visitor-type latrines. In 97 (21.09%) households, members defecated in the open air, and in 28 (6.09%), they defecated in a stream. The communes of Pissa (86.60%), Lessé (83.30%) and Mbaïki had the most latrines. Open defecation was frequent in the communes of Moboma (34.00%), Nola (30.80%), Baléloko (29.20%) and Mbata (26.00%). Of 330 households with a latrine, 185 (56.06%) were shared. A total of 271 (59.13%) households used soap for personal hygiene, compared with 168 (36.60%) who used no aseptic means for personal hygiene. The communes of Moboma (60.40%) and Bogongo-Gaza (59.30%) were the communes where the members of the households surveyed had no means of asepsis for personal hygiene. Of the 460 households surveyed, 442 (96.09%) were aware of the existence of a health facility. Of the 442 heads of household, 406 (91.85%) said they used health workers in the event of illness. In terms of accessibility, 326 (73.75%) households needed less than an hour to reach a health facility. However, the members of 114 (25.79%) households needed between 2 and 6 hours to reach a health facility. Table 2 shows the general characteristics of the households.

**Table 2:** general characteristics of the households surveyed

Variables	Number (n=460)	%
**Sex**		
Male	349	75.87
Female	111	24.13
**Type of population**		
Bantu	405	88.04
Aka	55	11.96
**Type of latrine**		
Visitor	296	64.35
Controlled open defecation	97	21.09
Open hole	30	6.52
Defecation in a stream	28	6.09
Other	9	1.96
**Presence and use of soap**		
Yes	271	58.91
No	168	36.52
Alternative	21	4.57
**Source of drinking water**		
Drilling	231	50.22
Unprotected water source	145	31.52
Unprotected well	44	9.57
Protected water source	25	5.43
Protected well	12	2.61
Other	3	0.65

### Participant characteristics

**General description:** a total of 1,967 people were examined, divided equally (50.00%) between men and women. Their mean age was 20.58±17.61 years, with extremes of 1 and 80 years. Children aged 1 to 14 years accounted for 52.92% of the total, or 1,009.

**Socio-demographic characteristics:** of the 1,967 people examined, 113 (5.74%) had skin lesions clinically suggestive of yaws. Men (n=71, 62.83%) were more affected than women (n=42, 37.17%), with a sex ratio of 1.69. The age of the participants ranged from 2 to 70 years, with a mean age of 26.69±19 years. Of the 25 children aged 6 to 14 with yaws, 22 (88.00%) were attending school. Of the 70 people over 14 with yaws, 63 (90.00%) were farmers, 44 (62.85%) were living common-law and 14 (20.00%) were single. Yaws was more common in people over 24 than under 25. In children under 10, yaws affected girls much more than boys. However, in people over 9 years of age, males were more affected than females. Table 3 gives a breakdown of people examined and suspected cases of yaws, by age group and sex.

**Table 3:** distribution of people examined and suspected cases of yaws by age group and sex

Age group in year	Number (n=1967)	%	Suspected case of yaws			%
			Male n(%)	Female n(%)	Total (n=113)	
1-5	411	20.90	8 (44.40)	10 (55.60)	18	4.38
6-9	295	15.00	5 (41.70)	7 (58.30)	12	4.07
10-14	303	15.40	9 (69.20)	4 (30.80)	13	4.29
15-24	277	14.10	8 (66.70)	4 (33.30)	12	4.33
25-34	239	12.20	9 (52.90)	8 (47.10)	17	7.11
35-44	183	9.30	14 (82.40)	3 (17.60)	17	9.29
45-54	129	6.60	6 (66.70)	3 (33.30)	9	6.98
55 et +	130	6.60	12 (80.00)	3 (20.00)	15	11.54

**Clinical and serological characteristics:** skin lesions due to yaws were multiple in 53.98% (n=61) of cases. These multiple lesions were seen more frequently in males (68.90%). The number of lesions was not associated with gender (p=0.07). Single lesions were more frequent (58.50%) in children aged 1 to 14 years. The number of lesions was significantly associated with age group (p = 0.01). Yaws lesions were papillomas in 76.10% (n=86) of cases. These papillomas were common in male individuals and in the age group of 1 to 14 years. Other types of lesions were uncommon. The seven cases of ulceration-type lesions were present only in children aged 1 to 14 years. The type of lesion was neither associated with age group (p = 0.06) nor sex (p = 0.185).

The preferred area for skin lesions was the lower limb in 74.33% of cases, and most often in males. Thoracic or abdominal lesions occurred in 80% of cases in females. Lesions localized to the head and face (12.40%) occurred in 92.90% of cases in children aged 1 to 14. Lesion location was significantly associated with age group (p = 0.04). Blood samples from the 113 suspected cases of yaws were all RPR-positive, 13 TDR-positive and 41 TPHA-positive. Taking into account our operational definition, the serological prevalence was 36.28%. Serological prevalence was highest in the communes of Nola (7.84%) and Baléloko (7.02%). In all, 18 (47.36%) of the 38 villages surveyed were endemic for yaws. Of the 9 communes in the Mbaïki health district, no cases of yaws were confirmed in the commune of Lessé. Finally, the prevalence of yaws was estimated at 2.08% in the Mbaïki health district. Table 4 below presents the different prevalences by commune in the district of Mbaïki.

**Table 4:** clinical, serological and yaws prevalences by commune

Communes	People examined	Suspected case of yaws	Clinical prevalence %	Confirmed case of yaws	Serological prevalence %	Yaws prevalence %
Mbaïki	124	5	4.03	3	60.00	2.42
Mbata	287	18	6.27	7	38.89	2.44
Pissa	243	13	5.35	5	38.46	2.06
Baléloko	285	20	7.02	4	20,00	1.40
Moboma	245	14	5.71	8	57.14	3.27
Nola	281	22	7.83	10	45.45	3.56
Bogongo-gaza	142	6	4.23	2	33.33	1.41
Lessé	132	8	6.06	0	0.00	0.00
Mongoumba	228	7	3.07	2	28.57	0.88
**HDM**	**1 967**	**113**	**5.74**	**41**	**36.28**	**2.08**

**Therapeutic characteristics:** of the 113 people with yaws-type lesions, 44 (38.93%) had received treatment. Among the 44 people treated, 14 (31.81%) had received benzathine benzylpenicillin and 29 (65.90%) had received other drugs whose names they did not know. Treatment costs ranged from 1,000 FCFA to 30,000 FCFA, with an average cost of 7542±7523 FCFA.

## Discussion

This study showed a yaws prevalence of 2.08%, a clinical prevalence of 5.74% and a serological prevalence of 36.28% in the Mbaïki health district. This result, which confirms the endemicity of yaws in the district, highlights the challenges of diagnosing skin lesions in general and yaws in particular. Indeed, several authors [10-14] have previously shown that yaws is endemic in the Central African Republic. Although the prevalence of 2.08% found in our series remains low compared with the prevalences found by Manirakiza *et al*. Boock et al or Hervé *et al*. [12-14], this difference in prevalence could be explained by the method used in our study. Indeed, in most of these studies, the method used consisted of exhaustive sampling with clinical examination of all community members present at the time of the surveys. The cluster sample survey used in our study could explain the differences in prevalences obtained. In addition, this method would better explain the variation of the disease in the population. This study differs from previous ones in its scope. Indeed, all the communes in the Mbaïki health district were affected, unlike other studies where only a few communes were targeted. This prevalence study, coupled with a sociodemographic and behavioral study, provides a better understanding of the causes of the disparity in the extent of the disease by commune, so that appropriate solutions can be found.

The study revealed that the communes of Nola, Baléloko and Mbata had higher prevalences of yaws, at 3.56%, 3.27% and 2.44% respectively. These high prevalences could be explained by the poor hygiene conditions observed in these communes. Yaws is known to be linked to poor hygiene conditions [2]. Indeed, in these communes, the population lacks adequate sources of drinking water, latrines and soap. These results are a reminder of the importance of hygiene and sanitation in strategies to control or eradicate yaws. Conversely, the prevalence obtained in our series is almost similar to those reported by Marks et al. in the Solomon Islands (1.10%) [16] and Ouédraogo *et al*. in Burkina Faso (1.55%) [17]. This could reinforce the reliability of our results. The serological prevalence of yaws is 36.28%. This rate is lower than the 50.00% reported by Widi-Wirski *et al*. [11]. The serological tests used to diagnose yaws are the same as those used to diagnose syphilis [18-20]. These tests use a non-specific treponemal antibody test (RPR) followed by a more specific treponemal test (TPHA) for confirmation. Serological confirmation in our study was based on RPR and TPHA tests. RPR tests are often positive in treated yaws, and TPHA is a specific treponemal serological test that remains positive for life [19,20]. This means that if one of our participants has, in the past, developed a Treponema pallidum infection, the test result will remain positive indefinitely and could therefore distort the diagnosis of yaws by its positivity. The combination of clinical examination and interpretation of serological results may reinforce the validity of our results.

The other observation is the high positivity rate of yaws lesions in Mbaïki and Moboma. Our study does not allow us to unequivocally explain these differences in positivity compared to the communes of Baléloko and Lessé. On the other hand, we observed in this study that the commune of Lessé had better hygiene and sanitation conditions, which could explain the absence of confirmation of yaws in this commune. However, it would be more judicious to carry out other more in-depth studies to research the factors linked to the transmission and persistence of yaws in the district to explain the observed phenomena.

The DPP test for screening and PCR for confirmation are the tests of choice for use in yaws eradication activities [21,22]. Given that we were unable to carry out these two types of test, it would be wiser to express some reservations regarding our results, especially as Ndzomo Ngono *et al*. in Cameroon have shown that certain skin lesions observed are not necessarily yaws lesions [23]. Similarly, almost 10 years ago, CAR joined the fight against trachoma by organizing mass distribution campaigns of azithromycin [24]; this may have implications for the small number of skin lesions observed in our sample, in contrast to the data published by Boock *et al*. [14], which show a clinical prevalence of 20.00% in the commune of Baléloko in Lobaye

In our series, all age groups were affected by this disease, with however, a predominance in the 55+ (11.54%), 35-44 (9.29%), 25-34 (7.11%) and 45-54 (6.98%) age groups; contrary to the results of some previous studies, which qualify yaws as a disease of childhood and adolescence [25]. The sex ratio of 1.69 found in our series confirms the findings that yaws affects males more frequently than females. Finally, most (89.86%) of the adults aged 15 and over with yaws-type lesions were farmers, and 62.32% were living common-law or were single (20.29%).

The study also revealed that few cases (38.93%) of skin lesions were managed. Even in those cases where skin lesions are managed, benzathine benzylpenicillin (32.93%) remains the most frequently used drug, in contrast to azithromycin (0.00%), which is recommended by the World Health Organization. The low rate of treatment of skin lesions could be explained by the high cost of treatment. Indeed, this cost varies from 1,000 to 30,000 FCFA; with an average cost of 7,500 FCFA; a cost far exceeding the daily budget of the majority of households in our study area. In the CAR, households spend an average of USD 2 per day, or around FCFA 1,000. For households, investing more than seven times their daily budget to solve a health problem that is not life-threatening is not a priority.

The non-use of azithromycin for the treatment of yaws-type skin lesions can be explained by health workers' lack of knowledge about yaws management, which in turn can be explained by the absence of a module on the management of NTDs in general, and yaws in particular, in health worker training curricula. Lastly, this study shows that the Mbaïki health district has the assets needed to effectively combat yaws. Indeed, the Mbaïki district is one of the country's districts with the highest rate of access to a water source and acceptable health service coverage, as 96.00% of households are aware of the existence of a health facility, and better still, over 95.00% of households need less than two hours to travel to a health facility.

## Conclusion

This study measured the extent of yaws in the Mbaïki health district. The prevalence of yaws, estimated at 2.08%, confirms the persistence of yaws transmission in the district. In the absence of implementation of the Morges strategy, the training of health workers in the diagnosis and management of yaws, and the provision of resources for the diagnosis and management of yaws, are means that can reduce the transmission of yaws, and contribute to the elimination of yaws in CAR and to the global objective of eradicating yaws.

### What is known about this topic

- *Yaws is targeted in the Neglected Tropical Diseases Roadmap 2021-2030 for eradication by 2030;*
- *Implementation of the Morges strategy is recommended by the World Health Organization for countries where yaws are endemic*.

### What this study adds

- *This study confirms the persistence of the yaws endemic in the Central African Republic;*
- *Contrary to other studies, it also reports that, in the Central African Republic, people aged 25 and over are more affected than those under 24, contrary to the idea that yaws is a disease of childhood and adolescence*.
